# The Golden Patient Initiative: A Systematic Review

**DOI:** 10.7759/cureus.39685

**Published:** 2023-05-30

**Authors:** Saad Khan, Bassil Azam, Abdulrahman Elbayouk, Alham Qureshi, Mobeen Qureshi, Adam Ali, Saif Hadi, Usman Ali Halim

**Affiliations:** 1 Trauma and Orthopaedics, Royal Oldham Hospital, Manchester, GBR; 2 Trauma and Orthopaedics, Maidstone and Tunbridge Wells NHS Trust, Tunbridge Wells, GBR; 3 Trauma and Orthopaedics, North Manchester General Hospital, Manchester, GBR; 4 Trauma and Orthopaedics, Royal Blackburn Hospital, Blackburn, GBR; 5 Trauma and Orthopaedics, Royal Bolton Hospital NHS Foundation Trust, Bolton, GBR; 6 Trauma and Orthopaedics, Hillingdon Hospital NHS Trust, London, GBR

**Keywords:** nhs, theatre efficiency, golden patient, systematic review, management of resources

## Abstract

Operating theatres and surgical resource consumption comprise a significant proportion of healthcare costs. Inefficiencies in theatre lists remain an important focus for cost management, along with reducing patient morbidity and mortality. With the emergence of the coronavirus disease 2019 (COVID-19) pandemic, the number of patients on theatre waiting lists has surged. Hence, there is a pressing need to utilise the already limited theatre time and fraught resources with innovative methods. In this systematic review, we discuss the Golden Patient Initiative (GPI), in which the first patient on the operating list is pre-assessed the day prior to surgery, and we aim to assess its impact and overall efficacy.

A literature search using the following four databases was conducted to identify and select all clinical research concerning the GPI: Medical Literature Analysis and Retrieval System Online (MEDLINE), Cumulative Index to Nursing and Allied Health Literature (CINAHL), Excerpta Medica Database (EMBASE), and the Cochrane library. Two independent authors screened articles against the eligibility criteria, using a process adapted from the Preferred Reporting Items for Systematic Reviews and Meta-analyses (PRISMA) guidelines. Data extracted included outcomes measured, follow-up period, and study design. The results showed significant heterogeneity, and hence a narrative review was conducted; 13 of the 73 eligible articles were included for analysis. Outcomes included delay in theatre start time, number of surgical case cancellations, and changes to total case numbers. Across the studies, a 19-30-minute improvement in theatre start time was reported (p<0.05), as well as a statistically significant decrease in case cancellations. Our analysis provides encouraging conclusions with regard to greater theatre efficiency following the application of GPI, a low-cost solution that can easily be implemented to help improve patient safety and lead to cost savings. However, at present, it is largely implemented among local trusts, and hence larger multi-centre studies are required to gather conclusive evidence about the efficacy of the initiative.

## Introduction and background

Surgical resource consumption accounts for over 30% of all healthcare costs [1]. Operating theatre inefficiencies, therefore, have far-reaching consequences. One-third of all National Health Service (NHS) operating lists suffer a delay of 30 minutes or more, with some estimates predicting potential daily savings of £3000 per hospital if these delays were halved [2-4]. Delays in surgery have long been understood to be strong predictors of increased patient morbidity and mortality [5-7]. Furthermore, a number of NHS trusts are having to cope with a significant patient and relative dissatisfaction, adding additional burden to the already struggling trust finances [8,9]. As a result, a consensus has emerged that optimising theatre efficiency is a paramount concern for NHS trusts.

The impact of the coronavirus disease 2019 (COVID-19) pandemic on surgical provision has exacerbated this problem. With staff redeployment and cancellation of elective surgeries, there has been a radical reduction in theatre capacity. An estimated 516,000 planned operations were cancelled in the UK during the initial wave of the pandemic [10], with a record 5.98 million patients waiting to receive treatment in England by October 2021 [11]. The proportion of individuals waiting more than 18 weeks for surgical treatment has doubled since the start of the pandemic to 34.4%. Although trauma services have continued to operate through the pandemic, the new safety measures implemented in hospitals to cope with the surge of COVID-19 infections have had a direct impact on theatre efficiency [12]. This significant rise in waiting times and surgical delays during the pandemic have uncovered an urgent need to investigate ways to improve theatre efficiency.

A number of strategies have been explored to improve theatre start times, case turnover, and overall theatre efficiency. These range from financial incentives [13], new protocols and systems [14], education [15], and improving overall team motivation [16]. Although these have shown promising results, their implementation requires capital and can often be more complex than initially thought. The lack of uptake of these strategies by NHS trusts reflects the challenges hospitals face in replicating them.

The term ‘Golden Patient’, first coined in 2013 by Javed et al., refers to a strategy where the first patient on the operating theatre list is pre-selected in advance, investigated, and prepared appropriately [17]. In doing so, delays to theatre start times are minimised, releasing greater theatre capacity. Since its inception, many trusts have adopted the Golden Patient Initiative (GPI) and reported favourable outcomes. However, there is a paucity of high-level data in the literature investigating improvements in theatre efficiency. In light of this, we aim to systematically review studies that have implemented the GPI, with the aim of assessing how the initiative impacts theatre efficiency.

This article was previously presented as a meeting abstract at the 2022 Annual Scientific Meeting of the Surgical Research Society on 24-25 March 2022.

## Review

Methods

This systematic review was conducted by adhering to the Preferred Reporting Items for Systematic Reviews and Meta-Analyses (PRISMA) statement for systematic reviews [18]. A systematic review design was chosen in order to maximise methodological rigour, reproducibility, and objectivity [19].

Eligibility Criteria

**Figure 1:**
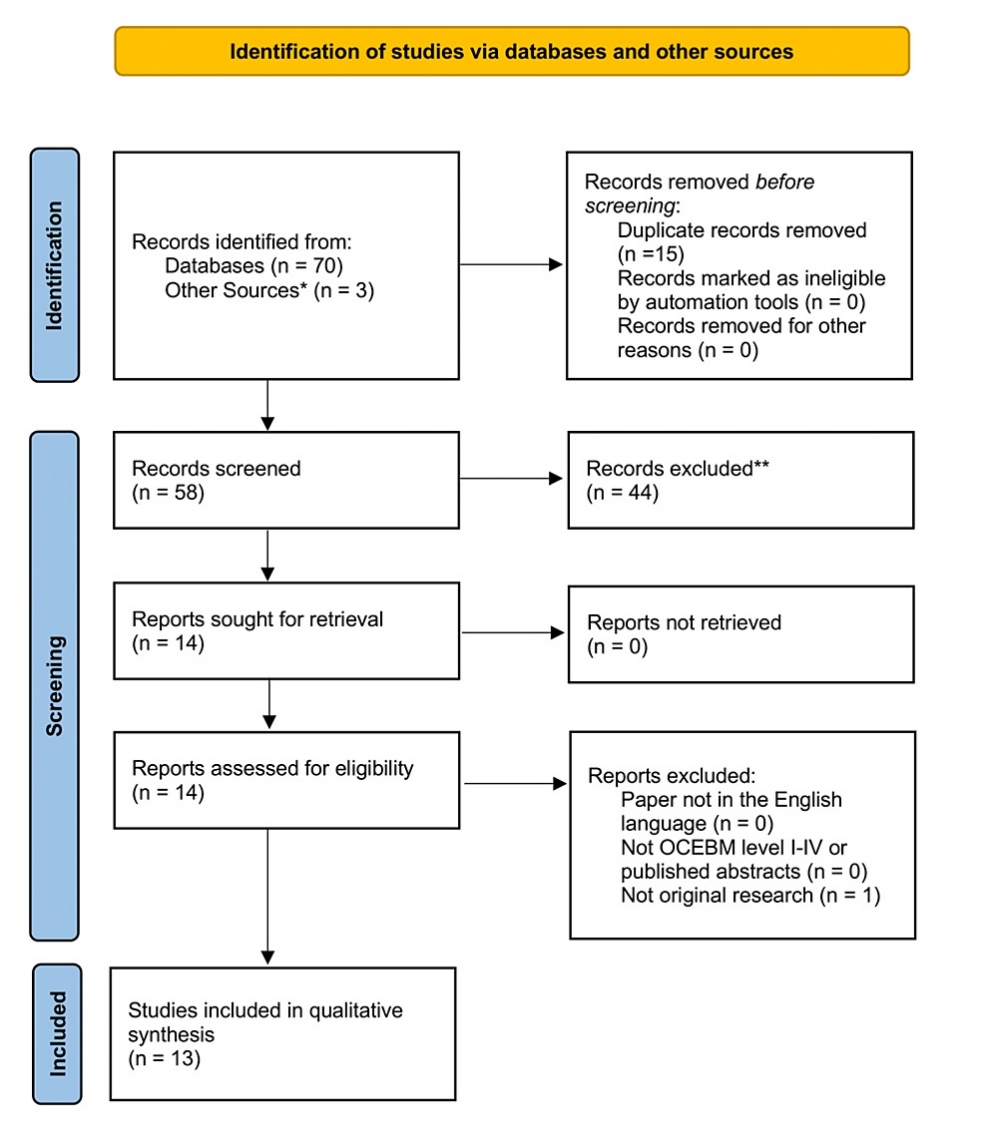
PRISMA flow chart depicting the selection of articles for review *Two additional records identified from the references of the full-text articles reviewed and search engines. **No implementation of the Golden Patient Initiative, or no measure of its effect PRISMA: Preferred Reporting Items for Systematic Reviews and Meta-analyses

Papers were required to meet several inclusion criteria, as shown in Figure 1. Firstly, they had to describe studies in which GPI was implemented and its effect measured. Secondly, they had to be written in the English language. Thirdly, they had to be classified as Oxford Centre for Evidence-Based Medicine (OCEBM) level 1 to 4, including peer-reviewed published conference abstracts. Lastly, the included papers had to describe an original research study. No restrictions were placed in terms of surgical speciality, study location, or date of publication.

Search Strategy and Information Sources

Medical Literature Analysis and Retrieval System Online (MEDLINE) from 1946 to May 2021, Emcare from 2005 to May 2021, Cumulative Index to Nursing and Allied Health Literature (CINAHL) from 1981 to May 2021, Excerpta Medica Database (EMBASE) from 1947 to May 2021 and the Cochrane Library databases were searched by using the following terms: golden AND patient AND [operat* OR room* OR theatre* OR list*] AND [efficien* OR start time*]. Duplicates were eliminated and the papers were then assessed for eligibility. This was conducted by two authors (SK and UH), who worked towards a consensus.

Data Extraction

Data were extracted from the eligible papers, including the lead author’s name, year of publication, surgical speciality, location of study, study design, follow-up period, and outcomes measured.

Statistical Analysis

The studies included in this systematic review displayed significant heterogeneity, in terms of surgical specialty, location of study, and outcome measures. As such, performing a meta-analysis of the data from these studies was deemed to be inappropriate, and thus a narrative review is presented.

Results

The application of the eligibility criteria resulted in the inclusion of 13 studies in the systematic review (Figure 1). A summary of the key findings from each of the 13 included studies is shown in Table 1. All studies were conducted in the United Kingdom in NHS hospitals. They were published during the period spanning 2013-2020. Four were full papers published in peer-reviewed journals, while nine were peer-reviewed conference abstracts. Seven studies observed the effect of GPI on orthopaedic trauma theatres, four looked at emergency theatres, one looked at neurosurgical theatres, and one looked into endovascular and cardiac catheterisation hybrid theatres.

**Table 1:** Summary of the studies eligible for inclusion in the systematic review GPI: the Golden Patient Initiative; QIP: quality improvement project; PDSA: Plan-Do-Study-Act

Author	Year and location	Study type and paper type	Sample size	Variables assessed	Results summary
Roberts et al. [9]	2015, Orthopaedic Trauma Theatre, George Eliot Hospital	Original research, audit	80 orthopaedic trauma cases	6 weeks follow-up (measurements taken from 6 weeks before intervention to 6 weeks after). Times: sent for, arrival in the anaesthetic room, arrival in theatre, the start of the operation, end of the operation, out of the theatre, and when next patient was sent for	Mean operation start time: 10:49 AM pre-intervention (CI: 10:43 to 10:55 AM), 9:50 AM (CI: 9:36 AM to 10:03 AM) = improvement of 59 minutes (p=0.00024)
Javed et al. [17]	2013, Orthopaedic Trauma Theatres, Royal Preston Hospital	Original research, prospective case-control	113 trauma lists	Mean operation start time (knife-to-skin), reception, anaesthetic and operation start times, number of cancelled operations	The mean reception time pre-GPI was 09:25; for the audited GPI period, it was 09:08; and for the actual GPI period, where the GPI remained first, was 09:01 (p<0.001) (improvement of 24 minutes). The mean anaesthetic start time pre-GPI was 09:35; for the audited GPI period, it was 09:16; and for the actual GPI period, it was 09:09 (p<0.001) (improvement of 26 minutes). The mean operation start time pre-GPI was 10:03; for the audited GPI period, it was 09:46; and for the actual GPI period, it was 09:33 (p<0.001) (improvement of 30 minutes). Reception, anaesthetic, and operation start times for pre-GPI data compared with lists where no GPI was selected were not statistically significant, thereby suggesting that the GPI is the cause of significance. The number of cancelled operations decreased significantly in the six months following the start of the study period. The number of cancellations totalled 49, and in the 6 months preceding the introduction of the GPI, the number of cancellations totalled 99 (p<0.01). The comparative mean reception times between weekdays and weekends for the audited period including the 95% CI for the population mean were also evaluated. The mean reception time for weekdays was 09:06, and for weekends, it was 09:14 (p=0.140)
Key et al. [20]	2019, Orthopaedic Trauma Theatres, Royal Gwent Hospital - Wales	Original research, QIP	3 sets of measurements were taken from the operating theatre; 1 month – baseline measurements pre-changes; 1 month – when “Golden Patient” was initiated (PDSA 1); 1 month – 4 months after the change (PDSA 2)	Orthopaedic trauma theatre – “operating capacity”. Time the patient reached the theatre. Knife-to-skin time. Finish time of list (both dressing on at end of case AND time patient left theatre). The number of cases completed. Total operating time for each procedure (knife-skin to dressing-on). On-the-day cancellation numbers and reasons	In PDSA2, there were 6 occasions where no Golden Patient had been identified. Three of these occurred over a weekend. Theatre start time and arrival in the suite improved from baseline to PDSA1 by a mean of −33 minutes (p≤0.001) and from baseline to PDSA2 by −29 minutes (p≤0.001). Procedure start time improved from baseline to PDSA1 by a mean of −19 minutes (p≤0.018) and from baseline to PDSA2 by −26 minutes (p≤0.001). Theatre finish time and procedure end time improved from baseline to PDSA1 by a mean of +53 minutes (p=0.124) and from baseline to PDSA2 by +52 minutes (p=0.126). Time out of theatre improved from baseline to PDSA1 by a mean of +48 minutes (p=0.169) and from baseline to PDSA2 by +44 minutes (p=0.2). Total operating time Improved by a mean of 16 minutes per list from baseline to PDSA1 (p=0.591) and 33 minutes per list from baseline to PDSA2 (p=0.104). Total cases: increase in total case numbers (baseline with 99 cases, PDSA1 with 119 cases, and PDSA2 with 135 cases). On-day cancellations: the total number of on-the-day case cancellations decreased (baseline: 26, PDSA1: 23 and PDSA2: 21)
Lobo and Harnett [21]	2019, Orthopaedic Trauma Theatres, King’s College Hospital	Abstract, audit	Unknown	3-week follow-up. Level of deviation from Golden Patient targets	Knife-to-skin time: 33 minutes later than the 9:00 am target time (SEM = 0.06): acceptable delay defined as 10 minutes. The needle-to-skin time: the target time of 8:30 am was never achieved; 24 reasons for delay noted: 33% of delays associated with needle-to-skin time in the Golden Patient
Shaw et al. [22]	2015, Endovascular Cardiac Catheterisation Hybrid Theatres, Guy’s and St Thomas NHS Trust	Abstract, original research, case-control study	N/A	Anaesthetic start time, theatre turnaround time (time between patient exit and start time of next operation), and patient throughput	The efficiency bundle reduced the theatre turnaround time by 20 minutes with a non-significant increase in throughput and fewer cancellations. A trend was observed towards earlier starts even on control days
Sumrien et al. [23]	2017, Emergency Theatre, Southmead Hospital	Abstract, QIP	N/A	Median theatre start time. Golden case identification	The median start time in the theatre was 8:20 AM in 3 days. Emergency theatre helped to clear patients from elective lists
MacKay et al. [24]	2018, Emergency Theatre, Glasgow Royal Infirmary	Abstract, QIP	Unknown	2-month follow-up. Timing of the first patient into the emergency theatre	GPI selection has further improved emergency theatre flow and, consequently, efficiency. Reduced late starts in emergency theatre % (from over 90% to just over 80%). GPI reduced late starts more than IHO and hub handover
Farooq et al. [25]	2013, Orthopaedic Trauma Theatre, Heatherwood and Wexham Park Hospital	Abstract, case-control study	35 trauma lists pre-Golden Patient and 35 post	2-month follow-up. Have beneficial items from Golden Patient guidelines been undertaken pre- and post-start of GPI? Theatre journey times	Patients with all items of the GPI checklist completed -65% pre-GPI introduction to 98% post. The mean time patient was sent for theatre: 8:43 pre to 8:20 post (p<0.001). The mean anaesthetic start time: 9:17 pre to 8:58 post (p<0.001). Post-GPI, 7 trauma lists added an extra case
Chauhan et al. [26]	2018, Emergency Theatre, Whipps Cross Hospital	Abstract, case-control	50 days	Selection of the first case, patient sending, and anaesthetic start times	The mean time for the first patient chosen improved from 08:13 to 05:39.(p=0.00047). The mean anaesthetic time improved from 09:27 to 09:02 (25 minutes) (p = 0.024)
Lee et al. [27]	2018, Orthopaedic Trauma Theatres, St George's University Hospital	Abstract, QIP	74 Golden Patients	Theatre call time and knife-to-skin time	47 of the 74 remained as Golden Patients, while 27 were no longer first on the list (either due to unsuitability or because other patients took clinical priority). Theatre call time improved by 24 minutes and knife-to-skin time improved by 15 minutes in Golden Patients compared to non-Golden Patients. However, compared to a previous audit at the same hospital, GPI had only improved knife-to-skin time by 3 minutes, suggesting that other factors affecting theatre start time had changed in the interim
Fernandes et al. [28]	2017, Orthopaedic Trauma Theatres, King's College London	Abstract, QIP	3 weeks	Delay in theatre start time (defined as the difference between the official time of 08:30 AM and the time into the anaesthetic room)	GPI decreased the delay in theatre start time by an average of 00:12:56 (to 00:30:35 from 00:43:13)
Tulloch et al. [29]	2020, Neurosurgical Theatres, King's College Hospital, London	Original research, QIP	24 weeks	Delay in theatre start time	Before GPI was implemented (Phase 1), there was an average delay of 86.7 minutes in starting theatre, and 91.7% of cases were delayed. The mean delay decreased by 72.3 minutes (p<0.0005) after GPI was introduced (Phase 2), and only 66.7% of cases were delayed. Breakdown of causes of delay: ward preparation and transfer to the theatre: 20% in Phase 1, 31% in Phase 2 (did not improve); preoperative documentation: 11% in Phase 1, 3% in Phase 2; investigations: 7% in Phase 1, 3% in Phase 2; medically unfit: 9% in Phase 1, 6% in Phase 2; surgeon availability: 5% in Phase 1, 10% in Phase 2; anaesthetist availability: 4% in Phase 1, 3% in Phase 2; theatre staff: 2% in Phase 1, 0% in Phase 2
McMonagle and Ramasaran [30]	2020, Emergency Theatre, Royal Liverpool and Broadgreen Hospitals	Abstract, QIP	80-90 patients	2-week follow-up. Patient fasting times (as an indicator of theatre delays). Theatre utilisation. Reasons for theatre delay. Golden Patient and fasting posters were combined as interventions	The mean fasting time decreased from 13 hours and 7 minutes to 11 hours and 0 minutes. The longest fasting time decreased from 43 hours to 41 hours. Prior to intervention: 3 patients began surgery before 9:30, and in 2 weeks post-intervention: 7 patients began surgery before 9:30

A range of different outcome measures were used across the included studies. Delay in theatre start time was the most commonly chosen parameter to determine GPI efficacy [9,17,20-29]. The number of theatre case cancellations was measured in three studies [17,20,22] while changes to total case numbers were also examined in three studies [20,22,25]. The effect on theatre end time was measured in two studies [9,20]. One study also assessed the impact of GPI on the turnaround time between cases. Other parameters recorded in single studies included total operating time [17], the time when the first patient on the list was chosen [26], and fasting time [30].

Theatre Start Time

Procedure start time was the most common variable recorded to measure theatre start time, with knife-to-skin time being defined as the start of the procedure in three studies. Key et al. recorded results in two sets: in the month the GPI was applied and four months after this [20]. They found that the procedure start time improved by a mean of 19 minutes (p≤0.018) in the first month and by 26 minutes (p≤0.001) in the second month. Javed et al. observed greater improvements; they investigated procedure start time six months after the GPI was implemented, in a major trauma centre carrying out 2200 trauma cases per year [17]. A mean improvement in operation start time of 30 minutes (p<0.001) was found.

Other studies also looked at procedure start time, without a description of how exactly the start time was defined. Roberts et al. analysed 80 orthopaedic trauma cases and found a large improvement in the mean procedure start time of 59 minutes post-GPI (p=0.00024) [9]. McMonagle and Ramasaran investigated the combined effect of both the introduction of the GPI and patient education posters on preoperative fasting [30]. They reported that theatre starts were often delayed, with three patients out of 80-90 in a two-week period undergoing surgery before 09:30, prior to their interventions. This increased to seven patients after intervention. The study also found that the mean fasting time decreased from 13 hours and seven minutes to 11 hours and 0 minutes.

Other measures of theatre start time were also utilised in a number of studies. Anaesthetic start time was used by Javed et al., which followed the same trend as the procedure start time with a 26-minute improvement (p<0.001). Shaw et al. investigated the effects of an efficiency bundle that included introducing a GPI, team briefings, and a joint surgical staff WhatsApp group [17,22]. A trend was displayed towards an earlier anaesthetic start time post-intervention, although this was not statistically significant. Farooq et al. collected data on 35 trauma lists over two months before introducing GPI, and 35 trauma lists after introducing the GPI [25]. It was found that the mean anaesthetic start time post-GPI was 19 minutes earlier (p<0.001). Chauhan et al. also measured the anaesthetic start time [26]. This study recorded results from an emergency theatre over 25 weekdays without the GPI and 25 weekdays once the GPI was introduced. A mean anaesthetic time improvement of 25 minutes was found (p=0.024).

Further outcomes representing theatre start time were additionally detailed in individual studies. These include the time of arrival in the suite recorded, with Key et al. showing an earlier mean arrival time of 33 minutes in the month GPI was initiated and 29 minutes four months after GPI [20]. Farooq et al. recorded a similar variable, demonstrating a reduction in the mean time that a patient was sent for of 23 minutes (p<0.001) [25]. Furthermore, Javed et a. reported a decrease in the mean time of arrival to theatre reception for the first patient of 24 minutes (p<0.001) [17].

Sumrien et al. conducted an audit on theatre efficiency and found that increasing the identification of a Golden Patient contributed to an improved median start time for the theatre of 8:20 AM, although the theatre start time was not defined [23]. Mackay et al. similarly conducted a quality improvement study to investigate the effects of multiple interventions on theatre efficiency [24]. In the first phase of this study, guidelines relating to handover from a quality improvement institute were implemented, besides commencing a multidisciplinary team handover on the mornings of emergency theatre lists. The GPI was introduced in the second phase, and results demonstrated a 10% decrease in late theatre starts over two months compared to phase 1.

Case Cancellations

A reduction in case cancellations was also exhibited across several studies. Key et al. reported a decrease in the total on-the-day cancellations measured across one-month periods [20]. Cancellations reduced from a baseline of 26 pre-GPI to 23 in the month after GPI was introduced, and to 21 four months post-GPI. This was despite an increase in total case numbers from 99 to 119 one-month post-GPI, with a further increase to 135 cases four months post-GPI. Javed et al. also reported a significant decrease (p<0.01) in cancellations, with 99 cancellations in the six months preceding the implementation of GPI compared to 49 cancellations in the six months post-GPI [17]. Shaw et al. also reported fewer cancellations and an increase in cases completed post-GPI, although this was non-significant and numerical values were not stated [22]. Additionally, Farooq et al. found that seven trauma lists post-GPI were able to add an extra case as a consequence of the GPI improving theatre efficiency [25].

Other Variables Recorded 

Supplementary data were also reported in some studies to assess the effect of the GPI. Key et al. reported non-significant improvements in the mean procedure end time of 52 minutes for the last case (p=0.126), and an earlier time out of the theatre by 44 minutes four months post-GPI (p=0.2) [20]. A mean improvement of 33 minutes per list in total operating time was also noted (p=0.104). Shaw et al. reported a reduction in theatre turnaround time of 20 minutes after the implementation of its efficiency bundle [22]. Chauhan et al. observed an improvement in the time for selection of the first patient before a morning list, with a change from a mean time of 08:13 pre-GPI to 05:39 post-GPI (p=0.00047) [26].

Javed et al. collected further data to assess whether GPI was the main factor contributing to improvements in theatre efficiency seen [17]. Theatre lists with no Golden Patient selected after the introduction of the GPI were compared with theatre lists before the GPI was introduced. There was no statistically significant difference found between procedure, anaesthetic, and reception start times pre-GPI compared to post-GPI when no Golden Patient was selected, and all other variables remained the same. Farooq et al. also noted a clear difference in the preparation of patients once the GPI was implemented [25]. They observed that 65% of items on the GPI checklist would be completed before GPI was introduced, compared with 98% post-GPI. Interestingly, Javed et al. also noted that mean reception times were eight minutes later on weekends than on weekdays (p=0.140) [17].

Lobo and Harnett discussed the importance of a number of protocols that are needed in order to maximise the efficacy of the GPI [21]. An audit was conducted over three weeks to investigate whether targets were still being met and GPI protocols followed in a unit that had been implementing the initiative. The study found that knife-to-skin times were delayed by a mean of 23 minutes after acceptable delay times, and needle-to-skin time was delayed past the target time in all cases. A total of 24 different reasons for these delays were noted. Key et al. also observed that there were six occasions in the cycle four months post-GPI where no Golden Patient was identified [20].

Discussion

This is the first systematic review investigating the effect of the GPI on theatre efficiency. Our review indicates that the GPI can improve theatre efficiency and provides favourable results when implemented across several different specialities and hospitals. Importantly, improvement was seen in theatre start time in all studies, and this was statistically significant in most cases across a range of metrics representing theatre start time. Furthermore, case cancellations also decreased in all studies that measured this variable. Data from Javed et al. also demonstrated that there was no statistically significant difference in theatre start time when comparing cases where a Golden Patient was not successfully selected after the start of the GPI, compared to a normal day pre-GPI implementation [17]. Improvement in a variety of other parameters has been demonstrated across studies, including theatre finish time, total operating time, total cases and fasting time [17,26].

Financial Implications

NHS trusts are impacted widely by late finishes in theatre lists, with data collected from 92 trusts showing over 30% of lists beginning 30 minutes late [31]. These late finishes may cause monetary losses and can affect staff morale [26]. GPI can also cut costs that are incurred when patients have increased waiting times for theatre, through a selection of the shortest and most urgent cases as well as through improved organisation of theatre lists. It has been shown that delays in surgeries from the time a patient enters the emergency department can result in significant costs and maybe a recurring issue at many trusts, with one reporting a third of patients waiting longer than 24 hours for emergency surgery [32]. Theatre utilisation has also been shown to be highly influenced by the size of operating lists and whether these overran, rather than the surgeon’s influence [33]. Accurate and deliberate list scheduling may be the key factor in running an efficient theatre list [34], a key variable that the GPI may be able to improve.

Alongside theatre start times, theatre turnaround time delays have been shown to cause losses of almost £1000 per theatre per day [4]. The largest cause for this delay was shown to be a delay in sending for patients, a factor that can be improved through the early planning that the GPI brings. Evidence has also shown that the GPI may result in greater time reductions for theatre cases compared with other interventions used [35]. This adds to the evidence that surgical units may benefit from implementing the GPI before other efficiency improvement initiatives.

Study Limitations

There was a lack of heterogeneity among studies, which made performing a meta-analysis unfeasible. The parameters measured differed between studies. For example, knife-to-skin time was used as a measure of procedure start time in three studies [17,20,21], while four studies [17,22,25-26] recorded anaesthetic start time. Procedure start time was also undefined in two studies [9,30]. This hindered the collective quantitative analysis of results from the papers. However, different theatre units may measure start times by different methods. The collection of data using a variety of definitions of the GPI make the results applicable to a larger number of surgical units. Furthermore, theatre start time alone may not always be a suitable representative of theatre efficiency [36] and this was a key variable used in the vast majority of included studies.

There was variation as to which days were used to record results. Chauhan et al. recorded results only on weekday lists, where staffing levels would be higher than on weekends [26]. Javed et al. highlighted this as a possible factor when finding that mean reception times were later on weekends compared with weekdays, despite GPI being implemented [17]. There was also variation in sampling methods between the studies, with many opting to record a set number of lists while others chose a specific number of days on which to collect data.

An improvement in communication may have been a confounding factor in these studies. As described in the protocol laid out by Javed et al., the selection of a Golden Patient requires communication between key members of the multidisciplinary team before the theatre list begins [17]. Communication itself has been shown to be a key factor in the effective running of theatre lists [37].

There was also some variation in the methods used to implement the GPI, which may affect reproducibility in new sites. An example of this is the time for the selection of the GPI patient. This was done at 8:30 PM on the night before by Javed et al. unless a life- or limb-threatening emergency was admitted overnight [17]. In contrast, Chauhan et al. reported a mean selection time for the GPI of 5:39 AM on the morning of surgery [26]. 

Limitations of the Golden Patient Initiative (GPI)

Selection of the GPI patient relies upon members of the surgical team assessing the urgency and the length of time of surgery before the list starts. This can vary and predictions may often be incorrect. In such cases, this impacts the remaining available theatre time, and thus may negatively impact theatre efficiency despite the selection of a Golden Patient [38].

Adherence is also an important factor in the long-term efficacy of the GPI. As seen by Key et al., arrival in the suite, procedure end time, and time out of theatre all showed greater improvements in the first month after the GPI, as compared to four months after the GPI [20]. In the cycle after four months, there were also six occasions where no Golden Patient was selected. Lobo et al. also demonstrated a lack of adherence to GPI targets in their audit, although other factors also contributed [21]. These results point towards the importance of regular auditing in order to keep the GPI effective over a longer period.

## Conclusions

This is the first systematic review investigating the effect of GPI on theatre efficiency. The analysis of results from studies showed improvement in theatre start time and reduction in case cancellation in all studies that measured these variables. Improvement in a variety of other parameters was also demonstrated across studies, including theatre finish time, total operating time, and total cases. Overall, GPI shows great promise for improving theatre efficiency, leading to greater patient safety and cost savings, which are much needed in the current post-pandemic era where waiting lists have increased exponentially.

However, there was a lack of heterogeneity in terms of parameters measured between studies, which made performing a meta-analysis unfeasible. Therefore, there is a need for multi-centre data providing further evidence of the benefits of GPI, in which there is uniformity in outcomes measured and study protocols. At this time, GPI can be seen as a low-cost, easily implementable solution that may bring benefits to many surgical units struggling to cope with theatre pressures.
